# Supplementary material for: Complexome profiling of the Chlamydomonas psb28 mutant reveals TEF5 as an early PSII assembly factor
Source: Plant Cell. 2025 Mar 17;37(6):koaf055. doi: 10.1093/plcell/koaf055 (PMC12164590; doi:10.1093/plcell/koaf055)
Supplement: koaf055_Supplementary_Data [file koaf055_supplementary_data.zip › psb28_interactive.zip!/plots/intensity/qualityPlots/peptides/nad5_Cre-2.g801492.t1.p1.html]

### Cre-2.g801492.t1.p1

Trivial name: nad5  
SeqCoverage [median]: 7.00  
Mass: 59.05 kDa  
  
 

| Sequence | MissedCleavage | Mass [Da] | wtIntensity | muIntensity | Score |
| --- | --- | --- | --- | --- | --- |
| GLLSVGNLR | 0 | 927.55 | 7683.91 | 8961.63 | 90.78 |
| GLLSVGNLRA | 1 | 998.59 | 20157.05 | 37674.09 | 114.50 |
| ITWAIGDR | 0 | 930.49 | 7039.44 | 2812.37 | 122.74 |
| SPVNPIFDLGR | 0 | 1213.65 | 35136.28 | 23424.70 | 180.15 |
| WGFDQLFAR | 0 | 1138.56 | 3309.50 | 0.00 | 109.86 |
|  |  |  | 73326.2 | 72872.8 |  |
